# Analyzing the interactions of mRNAs, miRNAs and lncRNAs to predict ceRNA networks in bovine cystic follicular granulosa cells

**DOI:** 10.3389/fvets.2022.1028867

**Published:** 2022-10-13

**Authors:** Kai Wang, Ying Cheng, Tong Guo, Xiangqian Guo, Hongzhi Zhang, Xiaoyan Ma, Yangyang Pan, Ermias Kebreab, Dong Wang, Lihua Lyu

**Affiliations:** ^1^College of Animal Science, Shanxi Agricultural University, Jinzhong, China; ^2^Department of Animal Husbandry and Veterinary Medicine, Beijing Vocational College of Agriculture, Beijing, China; ^3^Department of Animal Science, University of California, Davis, Davis, CA, United States; ^4^Institute of Animal Science, Chinese Academy of Agricultural Sciences, Beijing, China

**Keywords:** bovine, lncRNA, miRNA, ceRNA, follicular cysts

## Abstract

Cross-talk between competitive endogenous RNAs (ceRNAs) may play a critical role in revealing potential mechanism of bovine follicular cysts. Ovarian cyst has always been an intractable scientific problem and has led to considerable economic losses to bovine breeding industry. However, its pathogenesis and molecular mechanisms are still not well understood. Here, this study aimed to investigate the role of non-coding RNAs (ncRNAs) and the ceRNA networks in bovine follicular cyst. Whole transcriptome sequencing of bovine follicular granulosa cells (GCs) was conducted to obtain the expression profiles of mRNAs, lncRNAs and miRNAs. The results for the identified expressions of 8,003 mRNAs, 579 lncRNAs and 205 miRNAs were often altered between cystic and normal follicular GCs. Gene Ontology and Kyoto Encyclopedia of Genes and Genomes pathway analyses were performed on these differentially expressed mRNAs. Furthermore, the ceRNA network combining mRNAs, miRNAs, and lncRNAs using several bioinformatics methods based on co-expression analysis between the differentially expressed RNAs was conducted. Finally, the lncRNA NONBTAT027373.1-miR-664b-*HSD17B7* pathway was verified by dual-luciferase reporting assay and RNA binding protein immunoprecipitation (RIP) assay. LncRNA NONBTAT027373.1 sponged miR-664b in GCs and prevented miR-664b from binding to the *HSD17B7* 3′-UTR. These results indicated that genes and lncRNAs related to steroid hormone synthesis and energy metabolism could play important roles in the formation of bovine cystic follicles through the ceRNA mechanism and represent candidate targets for further research. This can be used as a practical guideline for promoting healthy and highly efficient development in the bovine industry.

## Introduction

The source of oocytes restricts animal reproduction and the development of gamete and embryo bioengineering. Only a few oocytes ovulate to fertilize and produce offspring during livestock reproduction (1). Domestic animals form oocyte repository with many primitive follicles during embryogenesis and shortly after birth (2). After birth, with the animal growth and development, the majority of follicles undergo atresia and degeneration (3). Post-puberty only a few follicles develop to mature, but the absolute number of follicles decreases with age (4). If female animal has ovarian disease, the limited resources cannot be used effectively, which causes significant losses to the breeding industry. Ovarian cyst is a common disease in livestock species, particularly in bovine (5). The issue increases with age, which seriously limits the effective utilization of bovine oocytes. The incidence of follicular cysts in bovine has declined since the advent of accurate diagnosis by B-ultrasound. However, under intensive breeding conditions, follicular cyst caused by feeding management and genetic breeding is still increasing gradually (6). Therefore, solving the occurrence of bovine follicular cysts can shorten calving interval, reduce the frequency of mating, improve bovine reproductive performance, and increase the economic benefits of the cattle farm.

Studies have been focused on the effects of hormones such as luteinizing hormone (LH), follicle stimulating hormone (FSH), estrogen (E_2_) and granulosa cell receptor proteins on the occurrence of follicular cysts (7). For example, recent study has elucidated that changes in the adrenergic system and the contractile force of the follicular wall may contribute to the development of follicular cysts (8, 9). The reduced number of granulosa cell layers in cystic follicles is associated with reduced expression of cell adhesion factors such as cadherin (cell-cell adhesion) and integrin (cell-extracellular matrix adhesion) (10). IGFBP3 is a key regulator of IGF-1 in animals with cystic follicles (11). Moreover, the decreased expression of ovarian matrix metallo proteinase 1/2 (MMP1/2) is an important marker of the occurrence of follicular cysts in cows (12). The low expression of BMP6 and GATA6 in GCs and the high level of intra-follicular lipopolysaccharide (LPS) play an important role in the formation of follicular cysts (13). Reduced hepatic 17β-estradiol gluconaldehyde and liver disease are also associated with the occurrence of follicular cysts in dairy cows (14). Local changes in some metabolic sensors in follicles produce an unfavorable microenvironment for the recovery of ovarian activity, leading to the persistence of follicles and the occurrence of follicular cysts (15). The early development of cystic follicles in dairy cows coincides with changes in the expression of some vascular endothelial growth factors (EGFs) (16).

mRNA has been the traditional research focus as a template for protein synthesis, while ncRNA has been regarded as a large number of byproducts of transcription with little biological significance (17). Since the 1950s, ribosomal RNA (rRNA), transport RNA (tRNA) and a variety of RNAs have appeared successively providing a new understanding of ncRNA (18). High-throughput sequencing technology has maximized the impact of ncRNA in the biological area. More than 90% of mammalian genomes are transcribed, of which only about 2% are involved in protein-coding functions (19, 20), the rest of the non-coding genomes are roughly divided into microRNAs (miRNAs, 18–22 nt), long non-coding RNAs (lncRNAs, 200–100,000 nt) and some ncRNAs of variable length. All these include circular RNA (circRNA), enhancer RNA (eRNA), and small interfering RNA (siRNA) etc., (21, 22). Although studies on ncRNAs are scarce, new breakthroughs have been achieved such as miRNA, lncRNA and circRNA since 2006. Studies have shown ncRNAs are involved in a variety of biological processes in humans and animals, including muscle growth (23), adipogenesis (24), fat deposition (25), and hair follicle regulation (26). In recent years, studies on ncRNA in bovine are mainly focused on the proliferation, differentiation and related signaling pathways of muscle cells (27), while the studies on reproduction, especially on gametogenesis are still lagging behind.

With the wide application of total transcriptome sequencing technology, ncRNA has been rapidly developed in the molecular biological area. Total transcriptome sequencing is considered as using high-throughput sequencing technology to detect RNA sequences, and the expression levels and differences of RNA are reflected to form a map of ncRNA expression (28). Specifically, total transcriptome sequencing is performed by building two libraries: 1 miRNA library and 1 rRNA chain-de-specific library (mRNA, lncRNA and circRNA). Sequence information of four types of RNA (miRNA, lncRNA, mRNA and circRNA) are obtained. GO enrichment, KEGG pathway and cluster analysis are performed on the expressive map. Finally, the regulatory relationship between ncRNA and coding RNA is integrated and analyzed, mainly including the interactions between the two: miRNA-lncRNA, miRNA-circRNA, miRNA-mRNA, and among the three: LncRNA-miRNA-mRNA and circRNA-miRNA-mRNA, in which the regulatory relationship (ceRNA regulatory network) is an important means to comprehensively analyze scientific problems at the molecular level. The ceRNA principle has been widely proved to be a common mechanism for the expression of nodular genes launching new research fields and resulting in the enhancement of the ceRNA network databases predicted by bioinformatics (29–32).

Nowadays, investigation of the occurrence of bovine follicular cysts is mainly concentrated in granulosa cell receptor protein and the negative balance of nutrition and energy (33). At the molecular level, studies on the genetic regulation mechanism of bovine follicular cysts, especially the molecular regulation mechanism of ncRNAs is relatively delayed. The lack to fully revealing the rules of the occurrence of bovine follicular cysts and the molecular genetic regulation mechanism seriously influences the exertion of bovine reproductive potential, and further restricts the application of bovine artificial insemination, OPU-IVF (34), G6G-timing sperm insemination (35) and B-ultrasound pregnancy diagnosis (36), and ultimately affects the development of bovine industry and the development of gamete (ovum) and embryo bioengineering. This study aimed to take the bovine follicular (normal and cyst) as the research object, obtain bovine granulosa cells (GCs) with high purity through cell biology techniques such as microanatomy and *in vitro* cell culture, and introduce total transcriptome sequencing technology to complete transcriptome sequencing of GCs. Based on the differentially expressed (DE) mRNAs and ncRNAs, the related ceRNA networks were constructed to identify the key genomes and related ncRNAs led to follicular cysts, and the related ncRNAs and the key genes led to the formation of follicular cysts were overexpressed and silenced at the cellular level through transfection and other molecular methods. In order to prove its regulation and target genes, the results are expected to identify the key genes and related ncRNAs mediated by clear ceRNA, further providing a scientific basis for understanding the genetic regulatory mechanism of bovine follicular cysts. It has important guiding significance for promoting the healthy and efficient development of bovine industry and gamete- and embryo-bioengineering.

## Materials and methods

### Ethical approval

All animal procedures were conducted according to the guidelines of the China Council on Animal Care. All experimental protocols were approved by the Ethics Committee of Shanxi Agricultural University [Permit NO: 2020(058)].

### Sample collection

Hereford cattle were raised under similar conditions, with free access to food and water and under natural lighting. Individual follicles were considered follicular cysts when the following symptom occurred: at least 25 mm in diameter with no luteal tissue found uni- or bilaterally in the ovaries detected by B-ultrasound successively for 7–10 d. The samples were obtained from Hereford cattle over 2 years of age, at least 45 days after calving (3.3 ± 1.5 lactations, range 2–7, mean 21 ± 5.5 kg of milk per day at diagnosis). Their ovaries were obtained after slaughter, cystic follicles and normal follicles were collected, which were transported to the laboratory in Dulbecco's phosphate-buffered saline solution containing antibiotics (100 IU/mL penicillin and 0.1 mg/mL streptomycin) at 4 °C. According to the selection criteria, 3 cystic (≥25 mm) follicles and 3 normal (10–15 mm) follicles were selected from different individuals. GCs from cystic and normal follicles were collected using microsurgery. After liquid nitrogen treatment, samples were placed in −80 °C freezer.

### RNA extraction

Total RNA was extracted from GCs using TRIzol RNA purification reagent, and genomic DNA was removed using RNase-free rDNase I (Takara, Japan), according to the manufacturer's instructions (Takara, Japan). The RNA quality was verified using the 2100 Bioanalyzer (Agilent, USA) and ND-2000 (NanoDrop Technologies). Only high-quality RNA samples (OD_260/280_ = 1.8–2.2, OD_260/230_ ≥ 2.0, RIN ≥8, 28S:18S ≥1.0, >10 μg) were used to construct a sequencing library.

### Library preparation and high-throughput sequencing

The longRNA-seq transcriptome library was prepared using the TruSeq total RNA kit (Illumina, San Diego, CA, USA) with 5 μg of total RNA. The ribosomal RNA (rRNA) was depleted instead of poly (A) purification using the Ribo-Zero Magnetic kit, which was then fragmented using fragmentation buffer. Next, random hexamer primers were used to synthesize first-strand cDNA. The RNA template was then removed, and a replacement strand was synthesized to replace dTTP with dUTP, thereby generating ds-cDNA. During polymerase amplification, the addition of dUTP quenched the second strand, preventing binding to the nucleotide. ds-cDNA was isolated from the second chain reaction mixture using AMPure XP beads. To prevent these blunt fragments from annealing together in the adapter ligation reaction, one “A” nucleotide was added to the 3′ end. Finally, the double-stranded cDNA was tagged with multiple indexing adapters. The library was 200–300 bp in size and amplified using 2% low range ultra-agarose, followed by amplification using 15 PCR cycles through Phusion DNA polymerase (NEB, Ipswich, MA, USA). After quantification using TBS380, the Illumina HiSeq XTEN //NovaSeq6000 (2 × 150 bp read length) was used to sequence the longRNA-seq library at the paired end. The sequencing adapter of the TruSeq Small RNA Sample Prep Kit (Illumina, USA) was also connected to 3 μg total RNA. The cDNA library was established by reverse transcription and PCR amplification using 12 PCR cycles. The deep sequencing was performed in Shanghai Majorbio Bio-Pharm Biotechnology Co. Ltd. (Shanghai, China).

### Read mapping and transcriptome assembly

The raw paired end reads were trimmed and quality controlled by SeqPrep (37) (https://github.com/jstjohn/SeqPrep) and Sickle (37) (https://github.com/najoshi/sickle) with default parameters. Then clean reads were separately aligned to reference genome (http://asia.ensembl.org/Bos_taurus/Info/Index) with orientation mode using HIASAT (38) (https://ccb.jhu.edu/software/hisat2/index.shtml) software. The mapped reads of each sample were assembled by StringTie (39) (https://ccb.jhu.edu/software/stringtie/index.shtml?t=example) in a reference-based approach.

### Differential expression analysis and functional enrichment

To identify DE transcripts (mRNAs, lncRNAs and miRNAs) between the normal and cystic follicular GCs, transcripts per million reads (TPM) was used to calculate expression of each transcript. RSEM Abundance values (http://deweylab.biostat.wisc.edu/rsem/) for quantitative genes were analyzed. The mRNAs, lncRNAs and miRNAs with a *p*-value ≤ 0.05 were considered DE transcripts by DESeq2 (40). GO (41) and KEGG (42) analyses were also conducted to determine which metabolic pathways and GO terms of differentially expressed genes (DEGs) were significantly enriched at Bonferroni-corrected *P* ≤ 0.05, compared to the whole transcriptome background. Goatools (https://github.com/tanghaibao/Goatools) and KOBAS (http://kobas.cbi.pku.edu.cn/home.do) were used to perform GO functional enrichment and KEGG pathway analyses, respectively.

### Analysis of ceRNAs regulatory network

We constructed a regulatory network of ncRNA and mRNA to uncover the underlying interaction mechanisms. Miranda (43) (http://www.miranda.org/) and TargetScan databases (http://www.targetscan.org/) were used to predict pairs of miRNAs with lncRNAs and mRNAs. Using the Pearson's correlation coefficient (44), the correlations between DE miRNAs and DE lncRNAs/DE mRNAs were evaluated, and those with *r* < −0.8 were selected for further analysis. Cytoscape (45) was used to build and visualize the interaction network.

### Real-Time quantitative PCR

RNAiso Plus reagent (Takara, Japan) was used to isolate RNA from normal and cystic follicular GCs. Then, 500 ng cDNA was extracted from the total RNA using PrimeScript™ RT kit, gDNA Eraser (Takara, Japan), and the housekeeping gene *ACTB* was used as an internal control for RT-qPCR amplification using TB Green Premix Ex TaqTM II (Takara, Japan) on a CFX96 real-time PCR system (Bio-Rad, USA). Briefly, the reaction was performed at 95 °C for 30 s, followed by 40 cycles of 95 °C for 5 s, 60 °C for 30 s. The first cDNA strand corresponding to miRNA was prepared using the M5 miRNA cDNA Synthesis Kit (Mei5bio, China) and amplified by RT-qPCR using the M5 miRNA qPCR Assay Kit (Mei5bio, China). The reaction was performed at 95 °C for 10 min, followed by 40 cycles of 95 °C for 15 s, 60 °C for 1 min. *U6* was considered as an internal control gene. Supplementary Table 1 shows the primer sequences synthesized by Sangon (Shanghai, China).

### RNA extraction from GCs nucleus and cytoplasm

Approximately 10^7^ GCs were required for the total number of cells. Paris^TM^ Kit (Invitrogen, USA) was used to extract GCs nuclear and cytoplasmic RNA, and RT-qPCR was conducted to analyze the expression of lncRNA NONBTAT027373.1 in the nucleus and cytoplasm. *GAPDH* and *U6* were considered as internal control genes.

### *In vitro* GCs culture

Serum-free long-term bovine follicular GCs culture medium containing Minimum Essential Medium (MEM) Alpha (1×; Gibco, USA) was configured, and HEPES (20 mM, Sigma, USA), sodium bicarbonate (10 mM, Sigma, USA), bovine serum albumin (1 g/L, Sigma, USA), penicillin-streptomycin liquid (100 IU/mL penicillin and 0.1 mg/mL streptomycin, Solarbio, China), MEM non-essential amino acids solution (1.1 mg/L, Gibco, USA), bovine insulin solution (10 μg/L, Sigma, USA), sodium selenite (4 μg/L, Sigma, USA), LR3-IGF-1 (1 μg/L, Sigma, USA), holo-transferrin (5 mg/L, Sigma, USA), androstenedione (10^−6^ M, Rhawn, China) and FSH (5 ng/mL, Solarbio, China) were added. GCs from those follicles with 10–15 mm diameter were washed with culture medium three times and resuspended in the medium. GCs were inoculated in 12-well cell culture plates (1 × 10^6^ viable cells/well) and cultured in a humidified atmosphere (5% CO_2_, 37 °C), with 75% medium replaced every 48 h with fresh medium.

### GCs transfection

Mimics, inhibitor and negative control (NC) of miR-664b, in addition to the siRNA of lncRNA NONBTAT027373.1, were designed and synthesized. The sequences are listed in Supplementary Table 2. The GCs were transfected using LipoFilter 3.0 (Hanbio, China), according to the manufacturer's instructions. A total of 1 × 10^6^ cells were inoculated into a 12-well plate containing complete medium, ensuring 60% confluence during transfection. We diluted 2.5 μL of 20 μM siRNA stock solution was diluted with 100 μL Opti-MEM and mixed gently. Opti-DMEM (100 μL) was added to the LipoFilter 3.0 reagent (3 μL) and incubated at 25 °C for 5 min. The mixture was gently mixed and incubated at 25 °C for 20 min before being added to each well. The plates were placed in an incubator at 37 °C with 5% CO_2_ for 48 h for subsequent experiments.

### Dual-luciferaes gene reporter assay

In a 12-well plate, GCs (1 × 10^6^ cells/well) were seeded and transfected. Using the ClonExpress II one-step cloning kit (Vazyme, China), the *HSD17B7* 3′-UTR/lncRNA NONBTAT027373.1 fragment complementary to the miR-664b seed region was subcloned into the pmirGLO plasmid digested with *Xho*I and *Sal*I to construct the wild-type (WT) plasmid. The mutant (MUT) plasmid was also constructed using the Mut Express II Fast Mutagenesis Kit V2 (Vazyme, China). All primer sequences are listed in Supplementary Table 3. miR-664b mimics/NC with recombinant plasmids were transfected into plasmid using LipoFilter 3.0 (Hanbio, China) according to the manufacturer's instructions. The luciferase activity was measured 48 h after transfection using a dual-luciferase reporter assay system (Promega, USA).

### RNA immunoprecipitation assay

The RIP assay was performed according to the following methods. Briefly, washed GCs (4 × 10^7^ cells) once with pre-chilled PBS buffer and collected the cells with a cell scraper into a 15 mL RNase-free centrifuge tube. Resuspended the cell pellet with immunoprecipitation lysis buffer (Beyotime, China) containing phenylmethylsulfonyl fluoride (PMSF) (BOSTER, China) and RNase inhibitors (Solarbio, China) and dissolved on ice for 30 min. Collected the lysate supernatant at 12,000 g/min for 20 min at 4 °C. Incubated A/G protein beads (MCE, USA) with Ago2 (dilution multiple 1:100) (BOSTER, China)/IgG (dilution multiple 1:100) antibody (ABclonal, China) overnight at 4 °C. The lysate was incubated with beads-antibody complexes at 4 °C for 6 h. The reaction mixture was centrifuged at 4 °C, 3,000 r/min for 2 min. Discarded the supernatant and washed the beads 5 times with RIP buffer (20 mM Tris-HCl, 150 mM NaCl, 1.5 mM MgCl_2_, 10% glycerol, 0.5% NP40, 0.5% Triton-X100). Incubated the beads with proteinase-containing k-eluate (Solarbio, China) for 45 min at 65 °C in a metal water bath. Took the supernatant, centrifuged at 4 °C, 3,000 r/min for 5 min. RNA was purified using chloroform-isoamyl alcohol (24:1) and precipitated overnight at −20 °C with glycogen (Solarbio, China). RNA was washed using 75% ethanol for 3 times, 10,000 r/min for 10 min at 4 °C. Thereafter, RNA was diluted in RNase-free water and the abundance of lncRNA NONBTAT027373.1 was analyzed by RT-qPCR.

### Statistical analysis

All experiments were repeated at least thrice. The data were analyzed by an independent samples *t*-test in SPSS 22.0, and were presented as mean ± standard error of mean (SEM). All data were normally distributed and the variance was similar between the groups. The relative expression of mRNA and ncRNA were calculated using the 2^−ΔΔCt^ method. A *P* < 0.05 indicated significant differences. A *P* < 0.01 indicated highly significant differences.

## Results

### Predictions and properties of non-coding RNAs in bovine GCs

**Figure 1:**
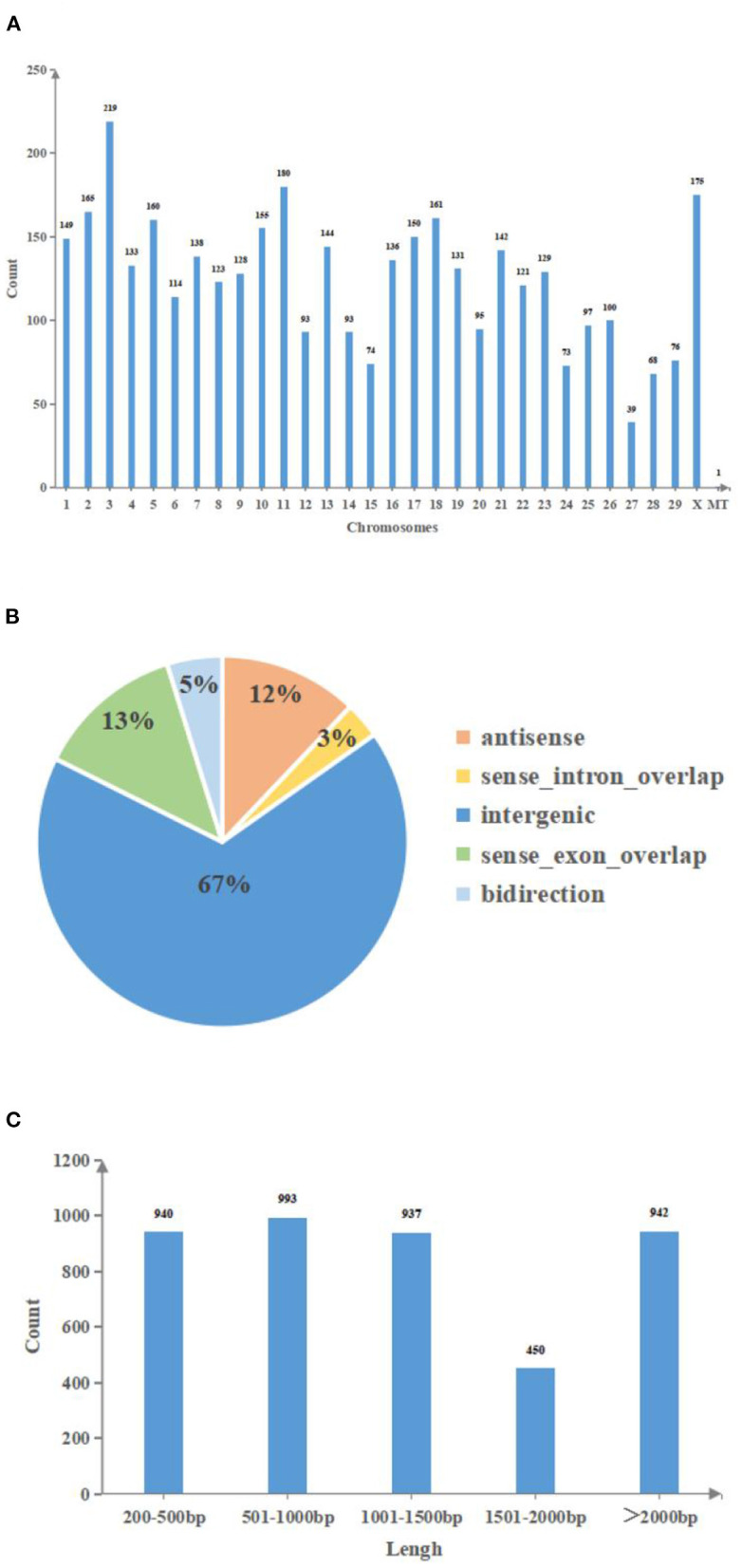
The features of lncRNAs in bovine follicular GCs. **(A)**: Chromosomes distribution of lncRNAs. **(B)**: Genomes distribution of lncRNAs. **(C)**: Length distribution of lncRNAs. MT, mitochondria; Count, Number of lncRNA transcripts; Chromosomes, Chromosome number; Antisense, lncRNA that overlaps with the antisense chain of coding genes; Sense intron overlap, lncRNA locates in the intronic region of coding genes; Intergenic, lncRNA locates in the intergenic region of coding genes; Sense exon overlap, lncRNA that overlaps with the exon region of coding genes; Bidirection, lncRNA locates in the 1 kb upstream region of coding genes, the transcription direction is opposite to coding genes; Lengh, The base number of lncRNA.

In a slaughterhouse, three cystic and three normal follicles were collected. Total RNA was extracted from qualified biospecimens and whole-transcriptome sequencing was performed. LongRNA-seq (lncRNA+mRNA) and smallRNA-seq (miRNA) data were obtained using an Illumina Hiseq2500 platform. After the redundant and low-quality reads were removed, the clean data obtained by longRNA-seq in all samples exceeded 14.9 GB, and the percentage of Q30 bases was >93.3% (Supplementary Tables 4, 5). Meanwhile, the raw reads obtained from smallRNA-seq in all samples exceeded 12.4 MB, and the percentage of Q30 bases was >95.3% (Supplementary Tables 6, 7). The datasets were submitted to the National Center of Biotechnology Information (accession number: PRJNA756563). In total, 3,443 lncRNAs and 1,282 miRNAs were identified on all chromosomes, and 2,667 lncRNAs were annotated in the bovine genome (Supplementary Table 8). Additionally, there was one lncRNA in mitochondrial DNA (mtDNA) (Figure 1A), and our data included intergenic, intronic, antisense and sense-overlapping lncRNAs. A majority (67%) of the lncRNAs were intergenic, while the minority (3%) were intronic (Figure 1B). The length of lncRNAs ranged from 66 to 26,123 bp. The majority (67%) of the lncRNAs were <1,500 bp (Figure 1C).

### Expression profiles of ncRNAs and mRNAs in bovine follicular cysts

**Figure 2:**
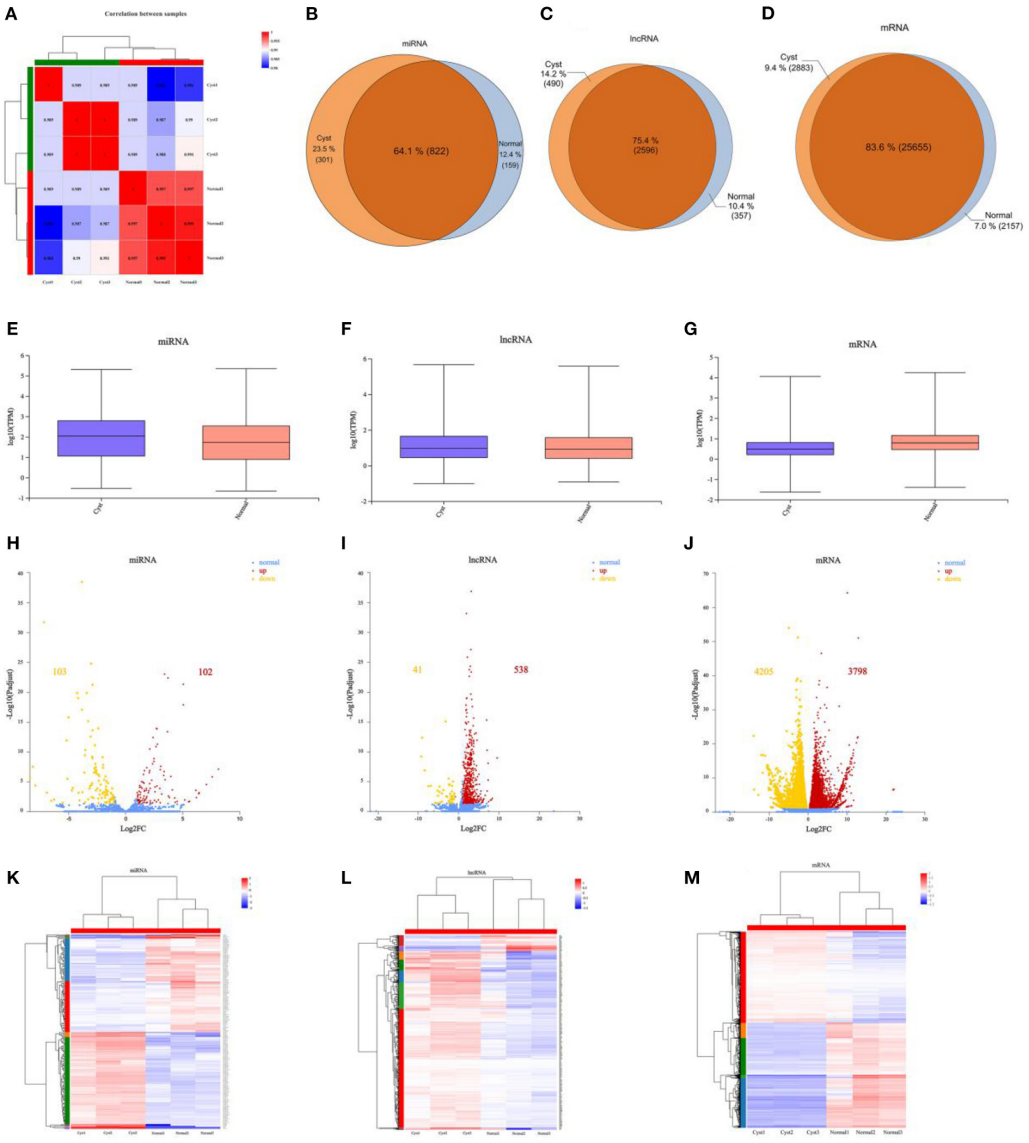
Comparative analysis of mRNAs, miRNAs and lncRNAs between normal and cystic follicular GCs. **(A)**: Correlation analysis between samples. **(B–D)**: The specific miRNAs, lncRNAs and mRNAs shared between normal and cystic follicular GCs. **(E–G)**: Boxplots showing the expression distribution of miRNAs, lncRNAs and mRNAs. **(H–J)**: Volcano plot of DE miRNAs, DE lncRNAs and DE mRNAs in normal and cystic follicular GCs. **(K–M)**: Hierarchical clustering of DE miRNAs, DE lncRNAs and DE mRNAs in normal and cystic follicular GCs. The red points showed the up-regulated miRNAs, lncRNAs and mRNAs, the yellow points showed the downregulated miRNAs, lncRNAs and mRNAs, and the blue points showed the equally expressed miRNAs, lncRNAs and mRNAs in volcano plots. Normal: Bovine ovarian follicles between 10–15 mm in diameter. Cyst: Bovine ovarian follicles greater than 25 mm in diameter.

The correlation between the selected samples were shown in Figure 2A and Supplementary Table 9. The DE ncRNAs (miRNAs and lncRNAs) and mRNAs in cystic and normal follicles were analyzed with cutoff fold changes ≤0.5 or ≥ 2, *P* < 0.05 and false discovery rate (FDR) <0.05 (Supplementary Tables 10–12). The results illustrated that 25,655 (83.6%) mRNAs, 2,596 (75.4%) lncRNAs and 822 (64.1%) miRNAs were shared by normal and cystic GCs (Figures 2B–D). The ncRNA and mRNA expressive abundances in bovine cystic follicular GCs were higher and lower than those in normal bovine follicular GCs, respectively (Figures 2E–G). DE mRNAs and ncRNAs in the samples are shown using a volcano plot (Figures 2H–J). The results elucidated there were 41 DE lncRNAs, 103 DE miRNAs and 4,205 DE mRNAs were downregulated in cystic follicular GCs compared with normal follicular GCs. In contrast, 538 DE lncRNAs, 102 DE miRNAs and 3,798 DE mRNAs were upregulated in cystic follicular GCs compared with normal follicular GCs. Hierarchical clustering of the DE ncRNAs and mRNAs revealed the expression patterns of the individuals for the same three comparisons (Figures 2K–M). We also discovered DE mRNAs and lncRNAs were distributed differently across the chromosomes (Supplementary Figure S1).

As shown in Supplementary Table 13, the most downregulated lncRNA was lnc-RAP2A with a 10^−3^-fold change, and the most upregulated lncRNA was XR_003036450.1, with a 764-fold change. The most upregulated mRNA and miRNA were *LRCH3* (4.3 × 10^6^-fold change) and bta-miR-184 (287-fold change), respectively. The most downregulated mRNA and miRNA were *ECE1* (6.39E-05-fold change) and bta-miR-33a (3 × 10^−3^-fold change), respectively. The top 10 downregulated and upregulated mRNAs and lncRNAs are described in Supplementary Tables 14, 15. The top five upregulated and downregulated miRNAs are listed in Supplementary Table 16. Six DE lncRNAs, six DE miRNAs and six DE mRNAs were randomly selected for this study. Reverse transcription quantitative PCR (RT-qPCR) was performed to detect the expression patterns of these transcripts in normal and cystic follicular GCs. The results were consistent with the RNA-seq data, indicating the reliability of the procedure (Supplementary Figure S2).

### Functional enrichment and construction of lncRNA-mRNA interaction network

**Figure 3:**
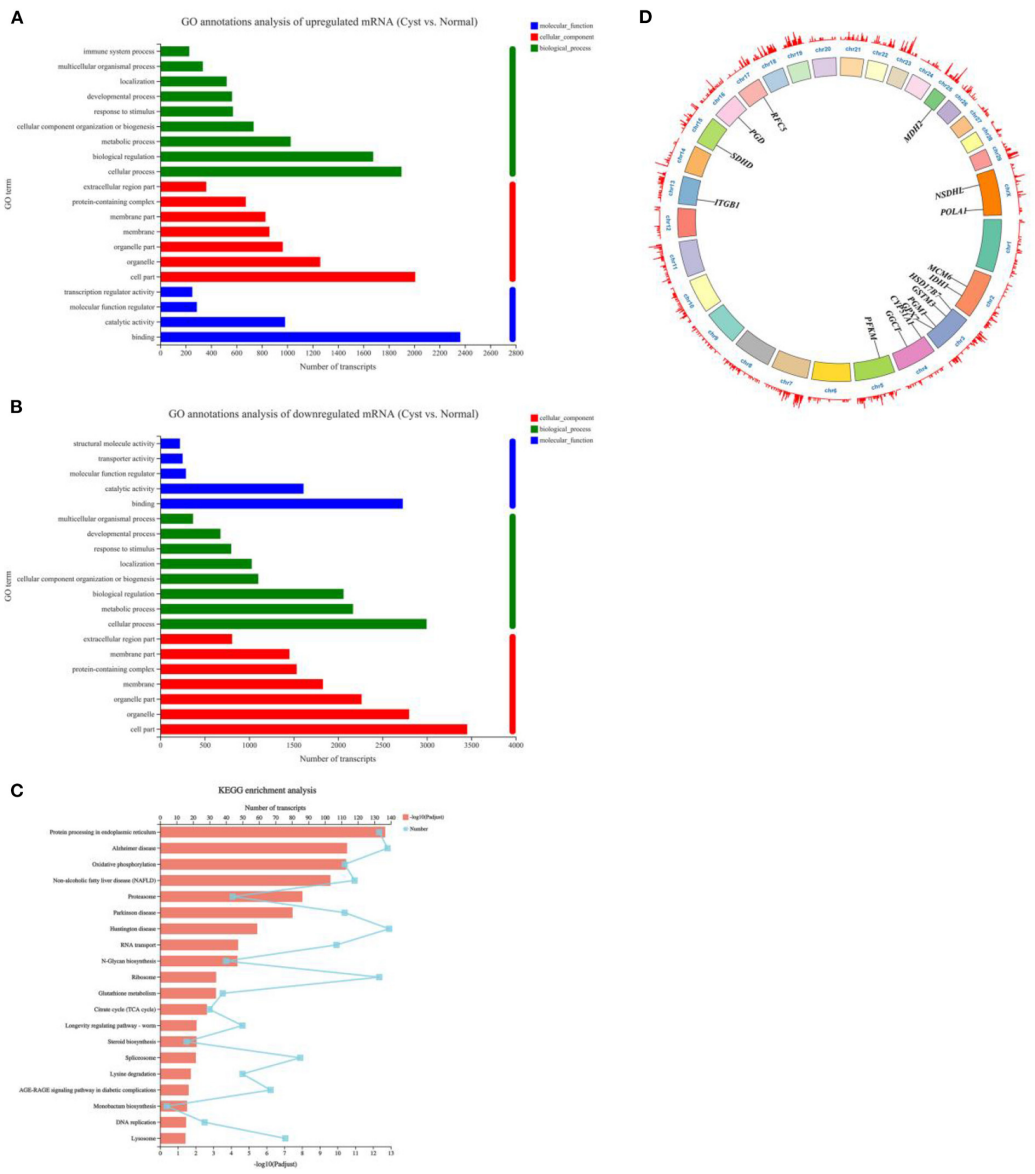
Functional analysis of DE mRNAs expressed in normal and cystic follicular GCs. **(A)**: GO analysis of DE mRNAs upregulated in bovine cystic follicular GCs; **(B)**: GO analysis of DE mRNAs downregulated in bovine cystic follicular GCs. **(C)** KEGG analysis. **(D)**: Circos plot of DE mRNAs associated with steroid hormone biosynthesis and metabolic (the inner ring represents 1 to 29 bovine autosomal and X sex-chromosomes. The outer ring indicates the chromosomes gene density).

Gene Ontology (GO) analysis revealed these DE mRNAs in bovine cystic follicles GCs were mainly related to binding (molecular function), cell part (cellular component) and cellular (biological process), and more DE mRNAs related to metabolic process (biological process) were downregulated in cystic follicular GCs, indicating abnormal metabolism of cystic follicles, which would affect the synthesis of cholesterol, the raw material of ovarian steroid hormones, as explained in Figures 3A,B. Kyoto Encyclopedia of Genes and Genomes (KEGG) pathway analysis revealed that multiple pathways were enriched in the DE mRNAs involved in bovine cystic follicular GCs. These genes were largely involved in steroid hormone biosynthesis pathways, oxidative phosphorylation, metabolic pathways, *etc*., all of which were important for follicular development (Supplementary Table 17; Figure 3C). Some DE mRNAs that could be associated with follicular cysts are shown in Figure 3D. Enrichment analyses of their target genes by GO and KEGG revealed the roles of DE lncRNAs. The 383 steroid hormone biosynthesis-associated DE lncRNAs were screened in the cystic vs. normal group (Supplementary Table 18). Furthermore, a co-expression network was constructed for the steroid hormone biosynthesis-associated DE lncRNAs (Supplementary Figure S3). This lays the foundation for investigating the potential function of lncRNAs in regulating target genes expression.

### Construction of ceRNA networks and characterization of candidate lncRNAs

**Figure 4:**
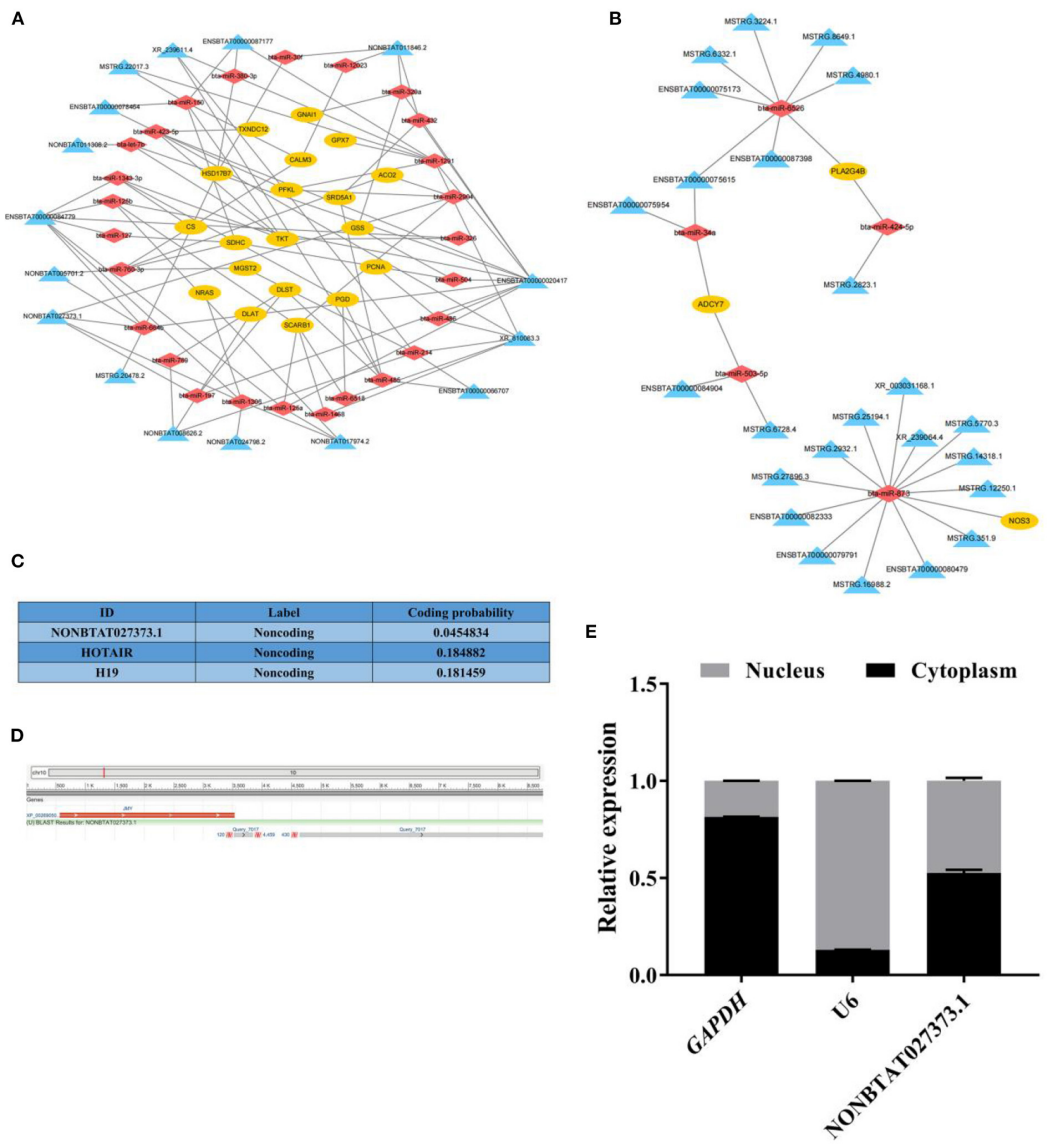
The network analysis of lncRNA-miRNA-mRNA and the basic information of lncRNA NONBTAT027373.1. **(A)**: The lncRNA-miRNA-mRNA networks of DE mRNAs downregulated in cystic follicular GCs. **(B)**: The lncRNA-miRNA-mRNA networks of DE mRNAs up-regulated in cystic follicular GCs. **(C)**: The coding potential of lncRNA NONBTAT027373.1 and other RNAs predicted using computational approaches. **(D)**: The sequence alignment of lncRNA NONBTAT027373.1. **(E)**: The expression of lncRNA NONBTAT027373.1 in GCs nucleus and cytoplasm. Yellow nodes represent mRNAs; red nodes represent miRNAs and blue nodes represent lncRNAs in the networks of lncRNA-miRNA-mRNA.

By the analysis strategy described in methods, the target mRNAs of these miRNAs were predicted based on miRanda and compared these target mRNAs with the DE mRNAs identified in the RNA-seq results. To fully explore the key genes related to follicular cysts and construct the networks more concise and effective, the key DE mRNAs closely related to bovine follicular cysts based on the literature and the results of KEGG pathway analysis in the above steps were further filtered. Then 22 mRNAs in the lncRNA-miRNA-mRNA networks were selected, which were involved in the nine pathways such as citrate cycle, estrogen signaling pathway, steroid biosynthesis, steroid hormone biosynthesis, ovarian steroidogenesis, DNA replication, pentose phosphate pathway, oxidative phosphorylation and glutathione metabolism. Then the lncRNA-miRNA-mRNA networks were constructed based on the selected mRNAs (Supplementary Table 19; Figures 4A,B). 115 lncRNA-miRNA-mRNA pathways were constructed including 40 lncRNAs, 31 miRNAs and 22 mRNAs. Among them, the expression levels of three mRNAs in cystic follicular GCs were significantly higher than that in normal follicular GCs, while the expression levels of 19 mRNAs in cystic follicular GCs were significantly lower than that in normal follicular GCs. These ceRNA regulatory relationships might be more important in the process of bovine follicular cysts formation considering the complexity among the ncRNAs and mRNAs.

In this study, the transcript NONBTAT027373.1 was obtained by longRNA-seq, and its sequence was identified as a known lncRNA with a total length of 4,889 nt by comparison with NONCODE database. The expression of this transcript in normal follicular GCs was significantly higher than that in cystic follicular GCs, which was a DE lncRNA. Coding potential analysis confirmed the non-coding nature of the transcript, similar to other well-characterized lncRNA, HOTAIR and H19 (Figure 4C). Furthermore, the transcript was located on the positive strand of DNA (location: 10381778-10387465) on bovine chromosome 10 and consisted of two exons, which overlap with the 3'-UTR of *JMY* (junction mediating and regulatory protein, p53 cofactor) (Figure 4D). This transcript was expressed in both the cytoplasm and nucleus of GCs (Figure 4E).

### Verification of the lncRNA NONBTAT027373.1-miR-664b-*HSD17B7* pathway

**Figure 5:**
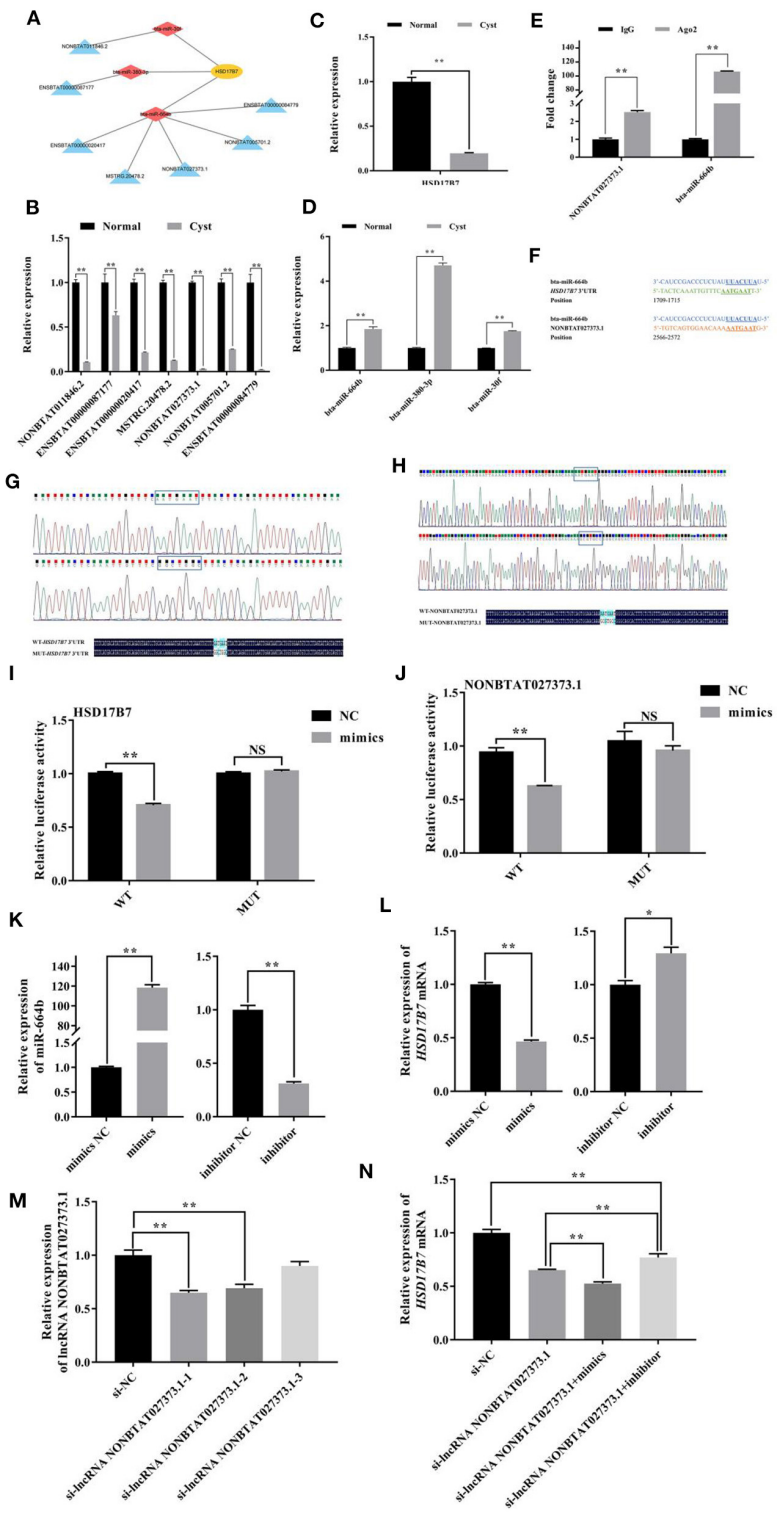
Analysis of miR-664b sponge HSD17B7 3'-UTR and lncRNA NONBTAT027373.1. **(A)**: The networks of lncRNA-miRNA-*HSD17B7* **(B)**: The expression of DE lncRNAs in lncRNA-miRNA-*HSD17B7* networks. **(C)**: The expression of *HSD17B7* mRNA. **(D)**: The expression of DE miRNAs in lncRNA-miRNA-*HSD17B7* networks. **(E)**: Interaction between lncRNA NONBTAT027373.1 and miR-664b through Ago2 protein investigated using the RIP assay. lncRNA NONBTAT027373.1 and miR-664b expression quantified by RT-qPCR. **(F)**: Schematic view of the miR-664b seed region, the motif of *HSD17B7* 3'-UTR and lncRNA NONBTAT027373.1. **(G,H)**: Construction of WT and MUT plasmid vectors of *HSD17B7* 3'-UTR and lncRNA NONBTAT027373.1. **(I)**: Dual-luciferase reporter gene analysis of the regulatory relationship between *HSD17B7* 3'-UTR and miR-664b. **(J)**: Dual-luciferase reporter gene analysis of the regulatory relationship between lncRNA NONBTAT027373.1 and miR-664b. **(K)**: Transfection efficiency of miR-664b after overexpression and knockdown of miR-664b. **(L)**: miR-664b regulates the expression of *HSD17B7* mRNA. **(M)**: RT-qPCR analysis of the effect on silence of lncRNA NONBTAT027373.1 expression by siRNA. **(N)**: Relative expression of *HSD17B7* mRNA in groups. NS, Non-significant. Values represent means ± SEM for three individuals. *P < 0.05, **P < 0.01. Yellow nodes represent *HSD17B7*; red nodes represent miRNAs and blue nodes represent lncRNAs in lncRNA-miRNA-*HSD17B7* networks. WT, Wild Type; MUT, Mutant; Mimics, Overexpression of miR-664b; Inhibitor, Knockdown of miR-664b; NC, Negative control.

KEGG analysis showed that HSD17B7 (hydroxysteroid 17-beta dehydrogenase 7) was a key enzyme in the steroid hormones synthesis of bovine ovarian, and its mRNA expression in cystic follicular GCs was significantly lower than that in normal follicular GCs. As shown in Figure 5A, in *HSD17B7*-related ceRNA networks, there were 11 nodes and 10 connections between one mRNA, three miRNAs and seven lncRNAs. The expression of lncRNAs, miRNAs and *HSD17B7* in the constructed ceRNA network were detected using RT-qPCR in normal and cystic follicular GCs (Figures 5B–D). The expression of miR-664b was increased in cystic follicular GCs. In contrast, the expression of its target genes including *HSD17B7* and lncRNA NONBTAT027373.1 were decreased. Moreover, an RNA-binding protein immunoprecipitation (RIP) assay revealed lncRNA NONBTAT027373.1 was enriched in the RNA-induced silencing complex miR-664b-Ago2. The enrichment of miR-664b and lncRNA NONBTAT027373.1 in the Ago2 group increased simultaneously, which was significantly higher than that in the IgG group, confirming the interaction between lncRNA NONBTAT027373.1 and miR-664b (Figure 5E). Software analysis demonstrated the miR-664b seed sequence was complementary to the *HSD17B7* 3'-UTR and lncRNA NONBTAT027373.1 (Figure 5F). Luciferase reporter plasmids containing the *HSD17B7*-WT and lncRNA NONBTAT027373.1-WT miRNA response element (MRE) motif or the MUT versions were constructed (Figures 5G,H). miR-664b significantly decreased luciferase activity of the reporter containing the WT-MRE motif, while the reporter activity of the MUT-MRE motif did not change (Figures 5I,J). These results indicated *HSD17B7* and lncRNA NONBTAT027373.1 could bind to the miR-664b seed region. In GCs, we also examined the transfection effects of miR-664b mimics and inhibitor (Figure 5K). Compared with the mimics NC group, the expression of *HSD17B7* was significantly downregulated after transfection with miR-664b mimics. In contrast, the expression of *HSD17B7* was significantly upregulated in GCs transfected with the miR-664b inhibitor (Figure 5L). The si-lncRNA NONBTAT027373.1-1 (termed si-lncRNA NONBTAT027373.1) with the highest inhibitory effect was selected for further investigation (Figure 5M). After transfecting si-lncRNA NONBTAT027373.1 into GCs, there was a significantly decrease in *HSD17B7* mRNA expression. However, co-transfection of miR-664b inhibitor and si-lncRNA NONBTAT027373.1 in GCs rescued *HSD17B7* mRNA expression (Figure 5N). We found that the miR-664b inhibitor could rescue the regulatory effect of si-lncRNA NONBTAT027373.1 on *HSD17B7*, suggesting that lncRNA NONBTAT027373.1 may regulate the function of GCs by sponging miR-664b-*HSD17B7*.

## Discussion

Ovarian cyst has always been a difficult scientific problem for the bovine industry. Therefore, its molecular mechanism is worth studying. In this study, mRNA, miRNA, and lncRNA expression profiles were compared in normal and cystic bovine follicular GCs to identify the key factors involved in bovine follicular cysts formation. In addition, we constructed and verified a potential lncRNA-miRNA-mRNA regulatory network, which provides new insights into the molecular mechanism of bovine follicular cysts formation.

When bovine follicular cysts, estrus is abnormal, estrus cycle becomes short, and estrus period is prolonged, or there is sustained and strong estrus phenomenon defined as nymphomania (46). Follicular cysts are single or multiple, in one or both ovaries, with diameter at least 2.5 cm (47). The ovarian wall is thin and dense, filled with cystic fluid, and slightly fluctuating. The inner granular layer is atrophy, the inner wall is only a layer of flat cells, and sometimes even disappears (48). Bovine follicular cysts most commonly occur on the 15–45 days of early postpartum lactation, an important stage in the transition from non-estrus to estrus cycle (49). Generally, its formation is due to hypothalamus-pituitary-ovarian axis dysfunction caused by multiple factors, including genetic, phenotypic, and environmental factors (50). The pathogenesis of follicular cysts is divided into primary defects at the hypothalamic-pituitary hydropin level and primary defects at the ovary-follicle level. However, the formation of follicular cysts is co-determined by both ovary-follicular and hypothalamic-pituitary defects (51). Affected hypothalamic-pituitary hormone release or altered follicle growth and development may result in abnormal hypothalamic-pituitary gonadal axis function (51). High milk production and the associated negative energy balance in the early postpartum period in cattle also lead to follicular cysts. During this time the energy demand maintaining milk production is higher than the energy intake, resulting in NEB, which is accompanied by a variety of hormonal and metabolic changes that affect ovarian function (52). Some animals can compensate for higher milk production by consuming more dry matter, thus reducing the impact of milk production on energy balance (53).

This study is the first to propose how mRNAs, pseudogenes, lncRNAs, and circular RNAs (circRNA) interact *via* microRNA (miRNA)-response elements (54). Many lncRNAs and circRNAs act as ceRNAs, sponging miRNAs and regulating mRNA expression, and cell differentiation in animals. For example, the ceRNA regulation network related to intramuscular fat deposition in yak was revealed through the whole-transcriptome landscape of muscle and adipose tissues to elucidate the function of genes associated with intramuscular fat deposition (55). RNA sequencing was used to identify ceRNA which may regulate lipid metabolism in bovine liver, and some key genes were found to affect lipid metabolism through insulin, PI3K-Akt, MAPK, AMPK, mTOR and PPAR signaling pathways (56). Studies have analyzed the differential expression patterns of mRNA, lncRNA, circRNA and their ceRNA networks in longissimus dorsi muscle of two different pig species (Chinese Huainan pigs and Western commercial Duroc pigs). A potential lncRNAs/circRNAs-miRNAs-mRNAs regulatory network was found to share MYOD1, PPARD, miR-423-5p and miR-874, which are associated with muscle proliferation, differentiation/regeneration and adipogenesis in skeletal muscle (57). A complete transcripome analysis of the toxic effects of zearalenone exposure on porcine GCs has been performed and a ceRNA network has been constructed. In addition, it was observed that ZEA exposure to the G2/M phase of porcine GCs blocked the process of cell cycle, and non-coding RNA (ncRNAs) played an important role in this process by regulating the expression of genes related to cell cycle arrest (58). The construction of ceRNA networks associated with skeletal muscle development in cattle revealed the co-expressed lncRNAs were significantly enriched in biological processes and Wnt signaling pathways associated with muscle development (59).

Recently, there have been some studies on mammalian ovarian ceRNA such as human, mouse and sheep, most of which involve lncRNAs. In addition, there are almost fewer studies related to ncRNA and ceRNA in bovine ovary. Recent studies suggest the mouse *Scd2* gene is activated by two lncRNAs and interdependent enhancers in ovarian follicular GCs, which provides a new insight into transcriptional activation in GCs (60). Studies have demonstrated FDNCR sponges miR-543-3p in GCs and prevents miR-543-3p from binding to the *DCN* 3′-UTR, resulting in *DCN* transactivation and TGF-β pathway inhibition and promotion of GC apoptosis in Hu sheep (61). *PWNR2* plays an important role in oocyte nuclear maturation in PCOS by functioning as a ceRNA to reduce the availability of miR-92b-3p for *TMEM120B* target binding during oocyte maturation in PCOS (62). lncRNA MALAT1 regulated apoptosis and estradiol synthesis of mouse GCs through the miR-205/CREB1 axis (63). The long non-coding RNA Xist regulates oocyte loss *via* suppressing miR-23b-3p/miR-29a-3p maturation and upregulating STX17 in perinatal mouse ovaries (64). Our findings simultaneously identified the lncRNA, miRNA and mRNA expression profile in the normal and the cystic follicular GCs by the whole transcriptome RNA-seq, there were 579 lncRNAs, 205 miRNAs and 8,003 mRNAs differentially expressed with 2-fold change.

In our study, some genes participated in steroid hormone biosynthesis, such as *HSD17B7* (hydroxysteroid 17-beta dehydrogenase 7), *NSDHL* (NAD(P) dependent steroid dehydrogenase-like) and *CYP51A1* (cytochrome P450, family 51, subfamily A, polypeptide 1), and the genes associated with pentose phosphate pathway such as *PGD* (phosphogluconate dehydrogenase), *PFKM* (phosphofructokinase, muscle) and *PGM1* (phosphoglucomutase 1). Meanwhile, in the GO analyses, the transition between the normal and cystic follicles for DE mRNAs was enriched. It has been well established energy metabolism is crucial to mammalian reproductive capacity, and the imbalance between nutrient intake and body storage during lactation is not sufficient to provide cholesterol as the raw material for steroid synthesis (65). NADPH+H produced by the pentose phosphate pathway provides a large amount of hydrogen for the synthesis of cholesterol and steroid hormones (66). Abnormal synthesis of steroid hormones, especially estrogen, may affect the feedback to the hypothalamus, leading to blocking synthesis of LH and failure to produce pre-ovulatory LH surge. Ultimately, ovulation fails, and the persistent existence of follicles causes follicular cysts (67). In addition, DEGs is also enriched in DNA replication, glutathione metabolism pathway and citrate cycle pathway. Therefore, the occurrence of follicular cysts could also be related to oxidative stress. The expression level of *ITGB1* (integrin subunit beta 1) in cystic follicular GCs was significantly lower than that of normal ones, and the low expression of *ITGB1* in GCs might be decreased cell-extracellular matrix adhesion (10) leading to the reduction of follicular granulosa cell layer and the decrease of estrogen level, which further affects ovulation and follicle persistence. *PGD* plays an important role in fat synthesis and metabolism in bovine adipocytes (68). Researchers have found that *PGD*, a cytoplasmic enzyme involved in carbohydrate metabolism, is necessary for the structural integrity of the endoplasmic reticulum (ER) and for protein secretion, and that *PGD* is the link between cytoplasmic carbohydrate metabolism and protein secretion. Moreover, chemical or genetic inhibition of *PGD* activity leads to cellular stress (69). The effects of different dietary protein and energy density levels on gene expression in adipose tissue of growing replacement lactating cows were studied using next-generation RNA sequencing technology (RNASeq), and the transcriptional regulation of energy metabolism of *PGD* was found and emphasized (70). In addition, phosphorylated *PGD* was found to be an important factor in the conversion of NADP+ to NAD+ (71). *PGD* is a key gene in the pentose phosphate pathway, which provides sufficient reduction equivalent for the synthesis of cholesterol, which is the raw material for the synthesis of steroid hormones, and the secretion level of steroid hormones affects the occurrence of bovine follicular cysts.

In the present study, we provided a systematic perspective on the potential function of ncRNAs in the bovine follicular cysts. The lncRNA-miRNA-mRNA regulatory networks in the bovine follicular cysts were comprehensively integrated and predicted on the basis of the screening and bioinformatics analysis. The mRNAs selected in the network conformed to the ceRNA rules and were also contained in known steroid hormone synthesis related genes or metabolic pathways. Then we constructed the lncRNA-miRNA-mRNA regulatory networks including 40 lncRNAs, 31 miRNAs and 22 mRNAs. Of them, there are 115 lncRNA-miRNA-mRNA pathways. These intertwined ceRNA networks suggests more important role in the follicular cysts regulation. In addition, to further demonstrate the credibility of our results, one predicted pathway, lncRNA NONBTAT027373.1/miR-664b/*HSD17B7* was selected and verified by the RT-qPCR, RIP and dual luciferase reporter gene system.

In fact, lncRNA NONBTAT027373.1 has not been studied yet. MicroRNA transcriptome analysis was conducted on the mammary glands of cows infected with *Staphylococcus aureus* and normal mammary glands, and miR-664b was differently expressed (72). This provided an experimental basis for revealing the etiology and regulatory mechanism of mastitis, and also suggested miR-664b can be used as a biomarker for the diagnosis of mastitis in cows (73). Studies have revealed miR-664b acts as a central regulator of gene expression in response to Gram-positive bacterial infection, and significantly regulates the sentinel ability of bovine mammary epithelial cells to mobilize the innate immune system (74). However, there has been fewer studies of miR-664b in bovine ovaries. The rodent (75) and human (76) HSD17B7 enzyme catalyzes the conversion of E_1_ and E_2_ *in vitro*, and the enzyme produced by E_2_ is important based on the high expression of *HSD7B7* in mouse ovaries. *HSD17B7* has a similar expression pattern with a known cholesterol stimulating enzyme during mouse embryonic development, suggesting a role for mouse *HSD17B7* in cholesterol biosynthesis (77). Analysis of cholesterol concentrations and various cholesterol biosynthesis intermediates in wild, heterozygous and *HSD17B7* KO mouse embryos clearly suggests *HSD17B7* is also essential for cholesterol biosynthesis in mice (78). In this study, we found lncRNA NONBTAT027373.1 and *HSD17B7* expression were simultaneously decreased, whereas miR-664b expression was increased in the cystic follicular GCs. Therefore, we speculated decreasing *HSD17B7* expression in bovine cyst follicles hindered the synthesis of steroid hormones in follicles and could not convert E_1_ to E_2_. The changes in the endocrine levels of sex hormones may cause the occurrence of follicular cysts. The validation of lncRNA NONBTAT027373.1-miR-664b-*HSD17B7* pathway lays a foundation for the study of ncRNA function in bovine follicular GCs.

## Conclusion

ncRNAs are involved in steroid hormone synthesis and energy metabolism in bovine follicular GCs, acting as ceRNAs for vital genes *via* functional miRNAs. Our findings provides a theoretical basis for further understanding the mechanism of ncRNA and ceRNA regulation in follicular development.

## Data availability statement

The original contributions presented in the study are publicly available. This data can be found here: NCBI, PRJNA756563.

## Ethics statement

The animal study was reviewed and approved by the National Institutes of Health.

## Author contributions

KW and LL conceived and designed the experiments. YC, TG, and XM collected samples. KW and YC drafted the manuscript. XG, YP, and HZ discussed and contributed to the data analysis. LL, DW, and EK revised the paper. All authors read and approved the final manuscript.

## Funding

This study was supported by Shanxi Youth Sanjin Talent Project, Shanxi Basic Research Plan Grant (Free Exploration, 20210302123393), Datong Key Research and Development (Agriculture) Grant (2021027) and the earmarked fund for Modern Agro-Industry Technology Research System in Shanxi Province.

## Conflict of interest

The authors declare that the research was conducted in the absence of any commercial or financial relationships that could be construed as a potential conflict of interest.

## Publisher's note

All claims expressed in this article are solely those of the authors and do not necessarily represent those of their affiliated organizations, or those of the publisher, the editors and the reviewers. Any product that may be evaluated in this article, or claim that may be made by its manufacturer, is not guaranteed or endorsed by the publisher.

## Abbreviations

BMP6: Bone morphogenetic protein 6
ceRNA: Competitive endogenous RNA
circRNA: Circular RNA
Cystic follicles: Bovine ovarian follicles >25 mm in diameter
CYP51A1, Cytochrome P450, family 51, subfamily A: polypeptide 1
CREB1: Cyclic AMP response element binding protein 1
DEGs: Differentially expressed genes
DCN: Decorin
E_2_: 17β-estradiol
E_1_: Estrone
ECE1: Endothelin converting enzyme 1
eRNA: enhancer RNA
FSH: Follicle-stimulating hormone
FC: Fold Change
FDR: False discovery rate
FDNCR: Follicular development-associated lncRNA
GO: Gene Ontology
GATA6: GATA binding protein 6
GCs: Granulosa cells
HSD17B7: Hydroxysteroid 17-beta dehydrogenase 7
IGFBP3: Insulin-like growth factor binding protein 3
IGF-1: Insulin-like growth factor 1
IVF: In-vitro fertilization
ITGB1: Integrin subunit beta 1
KEGG: Kyoto Encyclopedia of Genes and Genomes
lncRNA: Long non coding RNA
LH: Luteinizing hormone
LPS: Lipopolysaccharide
LRCH3: Leucine rich repeats and calponin homology domain containing 3
miRNA: Micro RNA
MMP1/2: Matrix metallo proteinase 1/2
mtRNA: Mitochondria RNA
MRE: miRNA response element
Normal follicles: Bovine ovarian follicles between 10–15 mm in diameter
ncRNA: Non-coding RNA
NEB: Negative energy balance
NSDHL: NAD(P) dependent steroid dehydrogenase-like
OPU: Ovum pick-up
PCC: Pearson correlation coefficient
PGD: Phosphogluconate dehydrogenase
PFKM: Phosphofructokinase muscle
PGM1: Phosphoglucomutase 1
RAP2A, RAP2A: member of RAS oncogene family
RIP: RNA-binding protein immunoprecipitation
siRNA: Small interfering RNA.
