# Supplementary material for: Analyzing the interactions of mRNAs, miRNAs and lncRNAs to predict ceRNA networks in bovine cystic follicular granulosa cells
Source: Front Vet Sci. 2022 Oct 13;9:1028867. doi: 10.3389/fvets.2022.1028867 (PMC9606814; doi:10.3389/fvets.2022.1028867)
Supplement: Supplementary file 2 [file Data_Sheet_2.docx]

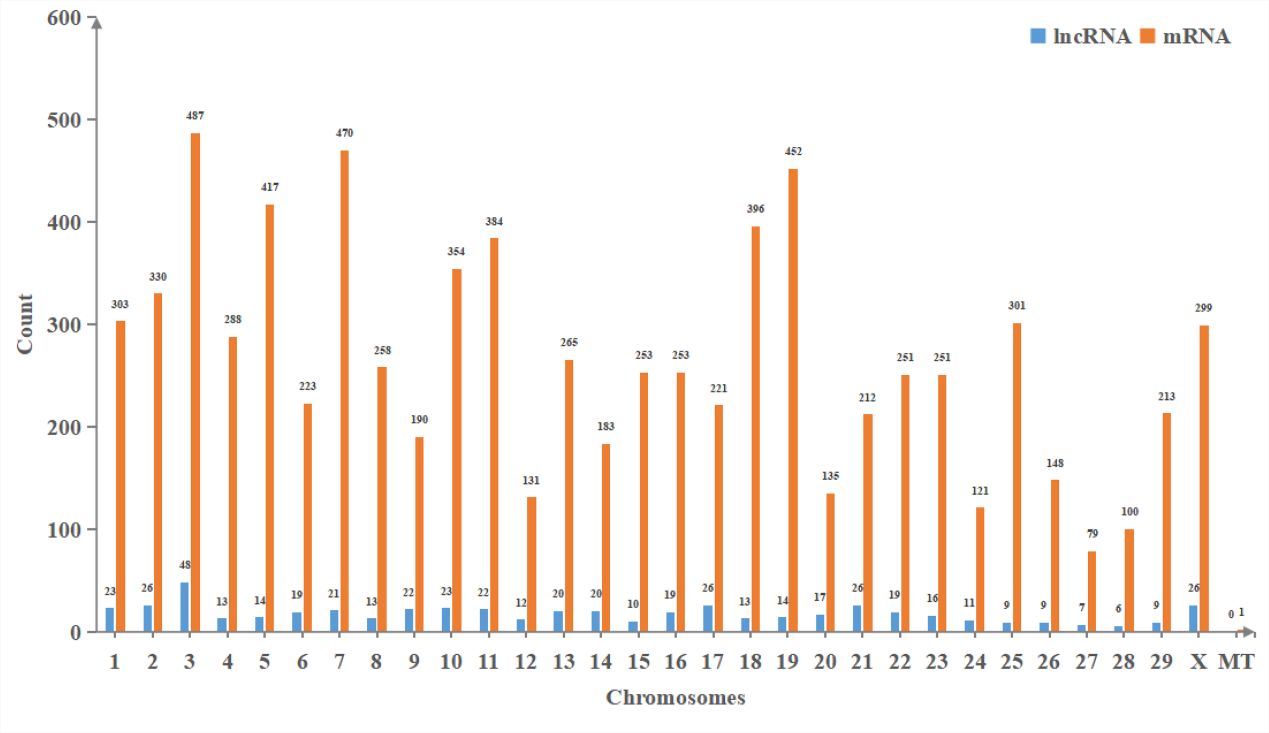


**Figure S1.** Comparison of lncRNA and mRNA distribution on chromosomes. **Notes:** MT: mitochondria. Count: Number of lncRNA and mRNA transcripts. Chromosomes: Chromosome number

**
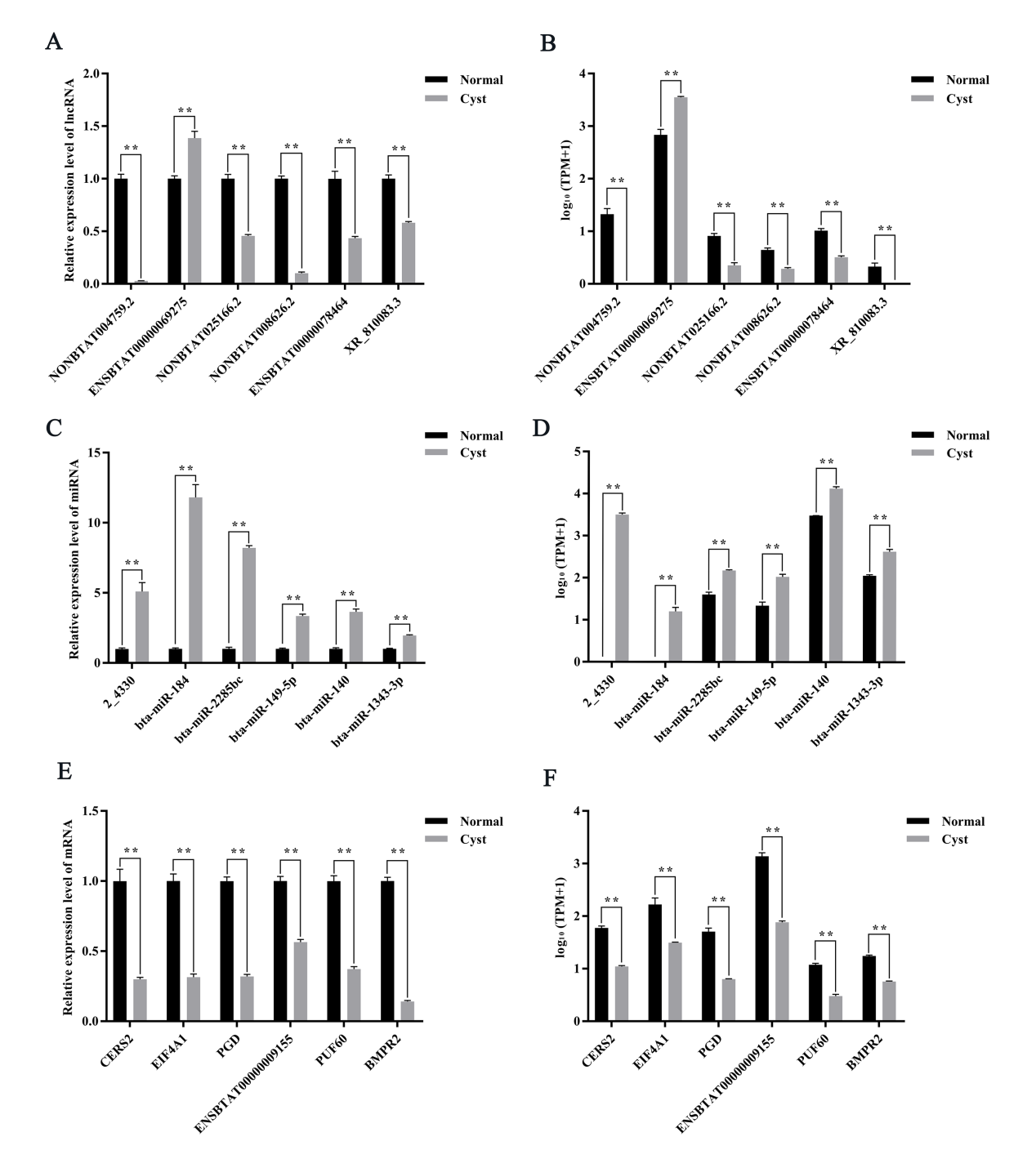
**

**Figure S2.** Verification of DE ncRNAs and DE mRNAs. **(A):** Validation of the DE lncRNAs using RT-qPCR. **(B):** Six lncRNAs randomly selected from the RNA-Seq data. **(C):** Validation of the DE miRNAs using RT-qPCR. **(D):** Six miRNAs randomly selected from the RNA-Seq data. **(E):** Validation of the DE mRNAs using RT-qPCR. **(F):** Six mRNAs randomly selected from the RNA-Seq data. **Notes:** The RT-qPCR data are elucidated as means ± SEM for three individuals. ***P*<0.01. The RNA-Seq data are presented as log_10_ (TPM+1) of each transcript. Normal: Bovine ovarian follicles greater than 25 mm in diameter. Cyst: Bovine ovarian follicles between 10–15 mm in diameter.


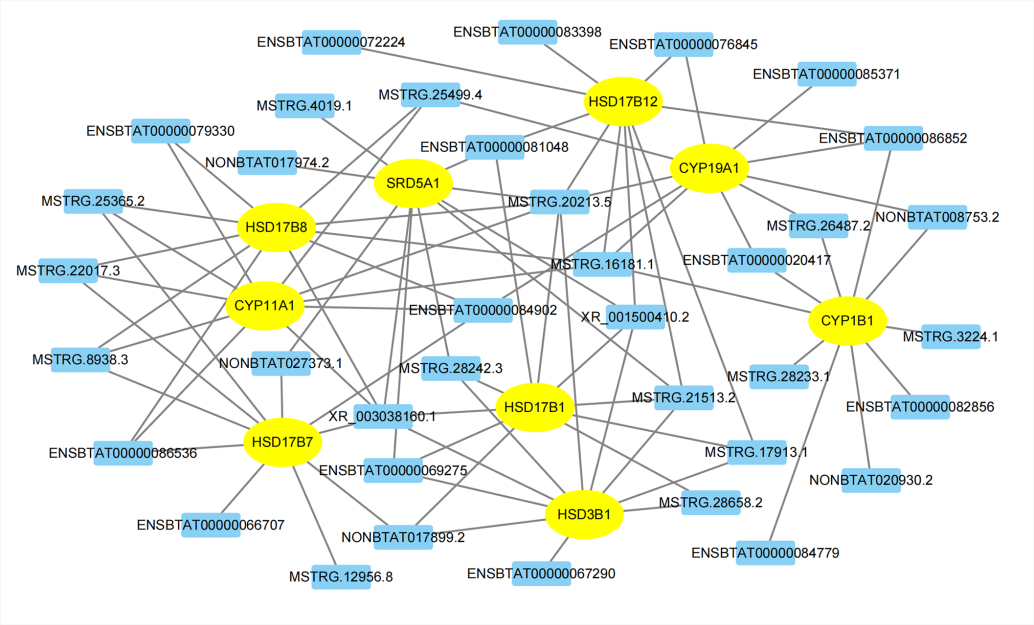


**Figure S3.** Co-expression networks of DE lncRNAs and steroid hormone synthesis related genes in GCs. **Notes:** Rectangle nodes represent lncRNAs and oval nodes represent mRNAs in the networks of lncRNA-mRNA.
